# Extracorporeal shock waves enhance normal fibroblast proliferation in vitro and activate mRNA expression for TGF-β1 and for collagen types I and III

**DOI:** 10.3109/17453670903316793

**Published:** 2009-10-01

**Authors:** Laura Berta, Annamaria Fazzari, Anna Maria Ficco, Patrizia Maurici Enrica, Maria Graziella Catalano, Roberto Frairia

**Affiliations:** ^1^Department of Clinical Pathophysiology, University of TurinTurinItaly

## Abstract

**Background and purpose** Extracorporeal shock waves (ESWs) are used to good effect in the treatment of soft tissue injuries, but the underlying mechanisms are still unknown. We therefore determined the effects of ESWs on normal fibroblasts in vitro, in order to assess treatment-induced cell response.

**Methods** A normal human fibroblast cell line (NHDF-12519) was treated with ESWs generated by a piezoelectric device (Piezoson 100; Richard Wolfe) using different protocols of impulses (300, 1,000, or 2,000 shots) and energy (0.11 or 0.22 mJ/mm^2^). Untreated controls and treated cells were cultivated for 12 days following a single shock-wave treatment. Viability, growth rate, and expression of mRNA for TGFβ -1 and collagen types I and III were evaluated at days 3, 6, 9, and 12.

**Results** 1 hour after shock-wave treatment, cell viability showed a decrease related mainly to impulse numbers applied. Fibroblasts treated with energy of 0.22 mJ/mm^2^ subsequently showed an increase in proliferation from day 6 to day 9 that was higher than in untreated controls, without interference with the normal cell kinetic profile. mRNA expression was also higher in treated fibroblasts than in untreated controls for TGFβ -1 on day 6 and day 9, for collagen type I on day 6, and for collagen type III on day 9.

**Interpretation** These in vitro data confirm that the main factors involved in the repair process of connective tissues are activated by ESWs. The study gives the rationale for, and may provide schedules for, ESW treatment of tendonopathies.

## Introduction

Fibroblasts play a crucial role in remodeling of the extracellular matrix by synthesizing and organizing connective tissue components. These cells are responsible for the synthesis and assembly of ECM molecules and their typical row orientation precedes collagen fibrillogenesis (Benjamin and Ralphs 2000). While collagen type I is the main component of collagen fibers, collagen type III has been shown to be important in the regulation of initial fibril assembly and thus at the early stages of injury repair (Birk and Mayne 1997).

Fibroblasts respond to various microenvironmental signals including soluble cytokines and growth factors, as well as cell matrix or cell-cell interactions that control the balance between synthesis and degradation of ECM (Atamas 2002). Mechanotransduction, i.e. the process of converting physical forces into biochemical signals subsequently integrated into a cellular response (Iqbal and Zaidi 2005, Vogel 2006), is of interest because many extracorporeal therapeutic devices use physical forces. Biosynthetic responses to physical energy (ultrasound, electromagnetic fields) observed in vitro and in vivo—increase in DNA synthesis, cell proliferation, extracellular matrix in bone and connective cells—Hsu and Chang (2004) have suggested the possibility of clinical use of this energy in bone and connective tissue repair.

Extracorporeal shock waves (ESWs) are acoustic waves that can induce a mechanical wave that passes through the cell compartment with cavitational effect; the cell response is proportional to the energy used (Martini et al. 2003). High-energy shock waves have been used mainly for the treatment of kidney, gall bladder, or salivary stones, while recently many researchers have applied shock waves of lower energy to injured soft tissues. In spite of improvements observed in a number of tendonopathies, the repair mechanism involved in shockwave treatment is still unknown. The fact that ESWs enhance both bone and tendon regeneration suggests that they may induce some form of signal for growth and maturation of the mesenchymal progenitors of bone marrow.

Wang et al. (2002) have shown that ESWs induce growth and differentiation of bone-marrow stromal cells via TGF-β1, but an activity involving membrane hyperpolarization with Ras activation and transcription factor CBFA1 expression has also been shown (Wang et al. 2001). Nitric oxide has also been suggested to mediate the anti-inflammatory effect of extracorporeal shock-wave treatment (Mariotto et al. 2005).

We explored the effects of ESWs on normal human fibroblasts in vitro and how the treatment can induce a cell response. We treated a normal fibroblast cell line in vitro with shock waves under different conditions of impulses and energy. After the treatment we evaluated fibroblast viability, the growth rate and pattern, and expression of mRNA for TGF-β1 and collagen types I and III—the main factors involved in the repair process.

## Material and methods

### Cell line and culture

The normal human dermal fibroblast cell line NHDF-12519 was purchased from Cambrex Bio Science (Milan, Italy) and cultured in accordance with the directions of the manufacturer. Briefly, cells were routinely maintained in 25 cm^2^ flasks at 37°C, in 5% CO_2_ with 95% humidity, in fibroblast basal medium (Cambrex Bio Science Walkersville Inc.) containing gentamicin and amphotericin (0.1%), insulin (0.1%) and hFGF-B (0.1%), and supplemented with 2% heat-inactivated FCS (Euroclone, Wetherby, UK).

### Exposure to shock waves

The shock-wave generator used for the in vitro experiments is a piezoelectric device (Piezoson 100; Richard Wolf, Knittlingen, Germany) especially designed for clinical use in orthopedics and traumatology. The instrument, which was kindly provided by Med and Sport 2000 (Torino, Italy), generates focused underwater shock waves at various frequencies (1–4 shocks per second) and at various intensities (0.05–1.48 mJ/mm^2^). The device comprises a high-voltage electric current generator and a reflector set in a water-filled container. On the surface of the reflector, piezoelectric elements arranged to form part of a sphere are stimulated with a high-energy electrical pulse. This causes vibration or rapid expansion of the crystals, leading to a shock wave that can be propagated through the water and focused at the center of the sphere. The pressure on the focal area is proportional to the voltage applied. The energy at the focal point is defined as the energy flux density (EFD) per impulse, recorded in joules per unit area (mJ/mm^2^). For use in orthopedics, shock waves of approximately 0.01–0.6 mJ/mm^2^ are applied (Martini et al. 2003). The focal area, which is peculiar to each kind of generator, is defined as the area in which 50% of the maximum energy is reached; with regard to the Piezoson 100 device, it has a length of 10 mm in the direction of the axis of the shock wave and a diameter of 2.5 mm perpendicular to this axis.

Aliquots (1 mL) of cell suspension adjusted to 1 × 10^6^ cells/mL were placed in 2-mL polypropylene tubes (Corning, New York, NY), which were then completely filled with culture medium. Subsequently, the cells were gently pelleted by centrifugation at 250 × g in order to minimize the motion during shock-wave treatments. The experimental set-up was as previously described (Frairia et al. 2003). Briefly, each tube containing cells was placed in vertical alignment with the focal area and was adjusted so that the central point of the focal area corresponded to the center of the bottom of the tube. The shock wave unit was kept in contact with the tube by means of a water-filled cushion. Common ultrasound gel was used as a contact medium between cushion and tube. Different ESW treatment regimens were investigated: (1) an EFD of 0.11 mJ/mm^2^ and peak positive pressure of 31 MPa (number of shots = 300, 1,000, and 2,000, respectively; frequency = 4 shocks per second), and (2) an EFD of 0.22 mJ/mm^2^ and peak positive pressure of 90 MPa (number of shots = 300, 1,000, and 2,000, respectively; frequency = 4 shocks per second). Cells that received no shock-wave treatment were used as controls. The cell viability following the HESW treatment was determined with trypan blue dye exclusion. A cell viability of 50–85% has been considered to be effective for evaluation of the subsequent cell growth rate.

Hydrophone measurements showed that peak pressure and pressure profile were only slightly altered inside the tubes (data not shown).

### Viability assay

After different treatment schedules, human fibroblasts from each tube were cultivated at a seeding concentration of 3,500 cells per well in quadruplicate, in a 96-well plate under standard culture conditions for 12 days. On days 3, 6, 9, and 12, the viability of treated and untreated cells was determined with an assay using 3-(4,5-dimethylthiazol)-2,5 diphenyl tetrazolium bromide (MTT; Sigma, St Louis, MO). Briefly, 10 μL of MTT was added to each well. After 4 h of incubation, 100 μL of 0.04 N NaCl in isopropanol was added and the absorbance at 495 nm was measured using a microplate reader (Model 680; Bio-Rad, Hercules, CA).

### Real-time PCR for TGF-β 1, collagen I and collagen III mRNA in normal human dermal fibroblasts

Total RNA was extracted from cells treated with ESW 0.22 mJ/mm^2^ (1,000 impulses) and untreated fibroblasts (control group) using TRIzol Reagent (Invitrogen, Groningen, the Netherlands) following the method developed by Chomczynski and Sacchi (1987). DNase I was added to remove remaining genomic DNA. 1 μg of total RNA was reverse-transcribed using the iScript cDNA Synthesis Kit (BioRad) following the manufacturer's protocol. Primers were designed using the Beacon Designer 5.0 program according to parameters outlined in the BioRad iCycler Manual. The specificity of the primers was confirmed by Blast analysis. The collagen I gene-specific primers used were as follows: forward 5'-TGG CAA AGA AGG CGG CAA AG, reverse 5'- AGC ACC AGC AGG ACC ATC AG. The collagen III gene-specific primers used were as follows: forward 5'-GAT GGT GCT CCT GGT AAG AAT GG, reverse 5'- GGG TCC TGT GTC TCC TTT GTC A. The TGF-β1 gene-specific primers used were as follows: forward 5'- ACT ACT ACG CCA AGG AGG TCA C, reverse 5'- AGA GCA ACA CGG GTT CAG GTA.

Real-time PCR was performed using a BioRad iQ Cycler Detection System with SYBR green fluorophore. Reactions were performed in a total volume of 25 μL including 12.5 μL of iQ SYBR Green Supermix (BioRad), 1 μL of each primer at 10 μM concentration, and 5 μL of the previously reverse-transcribed cDNA template. The protocol for primer sets was optimized using 7 serial 5× dilutions of template cDNA obtained from normal human dermal fibroblasts in basal condition. The cycling regime was as follows: denaturation (95°C for 5 min), followed by 40 cycles of 95°C for 15 seconds and 60°C for 1 min. A melt-curve analysis was performed after every run to ensure that there was a single amplified product for every reaction. All reactions were carried out at least in triplicate for every sample. The housekeeping gene used was that for β-actin.

### Statistics

Since the aims of our experimental study were to verify how different ESW treatments interfere with fibroblast cell growth and how expression of mRNA for TGFβ-1, collagen type I and III, is conditioned by experiment we chose as suitable for our goal, we analyzed the data by a statistical test performed on the average for paired samples and by an extension of the linear model of variance analysis i.e., the Generalized Linear Models (Dobson 1990). This statistical model was related to the experimental schedule, which included repeated measures over the time on every sample of: cells, gene expression for TGFβ-1, Collagen type I and III, respectively, then considered as the subjects of the statistical analysis. Thus, the within- and between-subject variabilities (Davidian and Giltinan 1995) were decomposed by a repeated-measures analysis of variance procedure.

Assumptions of normality and sphericity were tested by the Kolmogorov-Smirnov and Mauchly tests, respectively. Levene's test was used to test the variance equality; when sphericity could not be assumed, the Greenhouse-Geisser, Huynh-Feldt, and Lower-bound corrections were considered. The data were grouped as follows: (1) data related to different ESW treatments; and (2) data related to TGF-β1, collagen type I and collagen type III gene expression: the variables considered were treatment and interaction time/treatment.

Within- and between-subjects effects were considered significant with p-values less than 0.05. The statistical analyses were done using SPSS 13.0 for Windows.

## Results

### Cell viability

**Figure 1.**
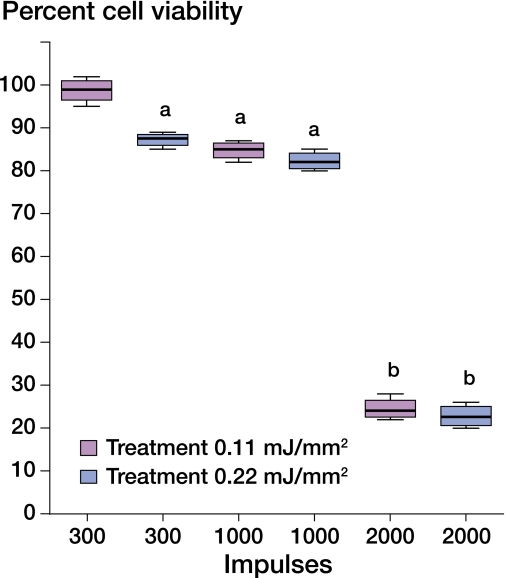
Effect of ESW treatment on cell viability. Viability is expressed as ratio between cells treated with shock waves and untreated controls (n = 16). **^a^** p < 0.05; **^b^** p < 0.001 relative to untreated controls.

1 hour after the shock-wave treatment, the cell viability showed an apparent decrease related both to the energy and the number of impulses applied: a constant decrease was observed in relation to the number of impulses (300, 1,000, 2,000) with a maximum reduction in viability at 2,000 impulses (viability 18%) while there was no statistically significant association between energy levels (0.11 and 0.22 mJ/mm^2^) and fibroblast viability (Figure 1).

### Cell growth pattern

**Figure 2.**
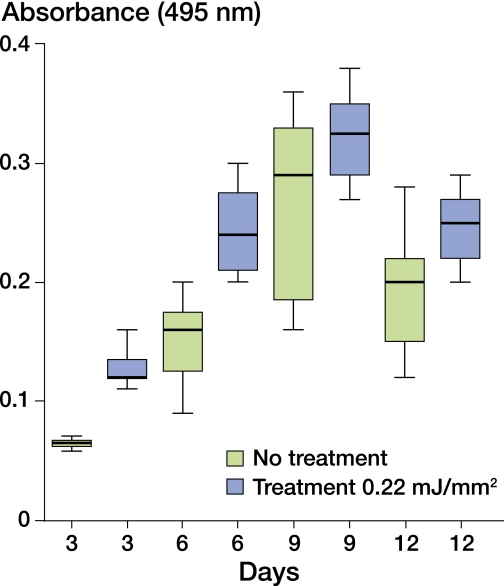
Effect of ESW treatment on cell growth. Cell growth was determined by the MTT method. Day 0 was seeding day (n = 16).

Fibroblasts treated with ESW (energy = 0.22 mJ/mm^2^; 1,000 and 2,000 impulses) showed a significant increase in cell growth relative to the controls (p < 0.001). A critical increase in cell growth was observed from the sixth to the twelvth day of the proliferation curve (Figure 2). No change was observed in the pattern of the growth curve either in treated or in untreated cells (p > 0.05). Since the goal of ESW treatment of tendon lesions is to promote and improve the repair process, treatment at the 0.22 mJ/mm^2^ energy level with 1,000 impulses appears to be the condition in which fibroblast viability fits growth dynamics.

### RNA expression

**Figure 3.**
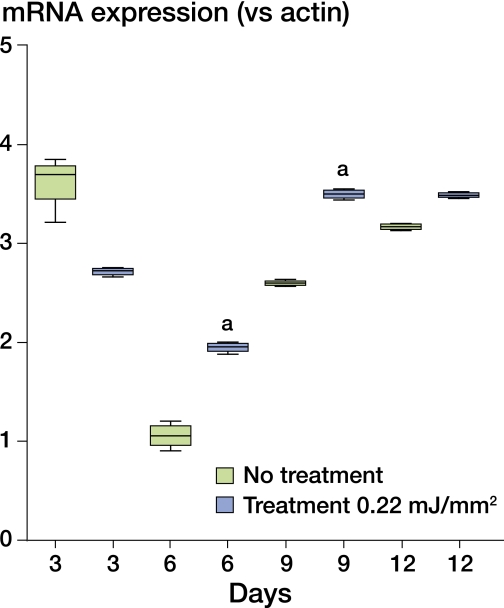
Effect of ESW treatment on TGF−β1 expression. Relative expression of TGF−β1 after treatment with ESW (EFD = 0.22 mJ/mm^2^, 1,000 impulses) (n = 4). Significance compared to no treatment: **^a^** p < 0.05.

The pattern of expression of TGF-β1 mRNA was similar in ESW-treated and untreated fibroblasts over the period of observation. A decrease in expression was observed in both groups from the third to the sixth day (p < 0.001). The following increase showed higher values in treated fibroblasts than in untreated fibroblasts for the sixth (p = 0.02) and ninth days (p = 0.02), respectively (Figure 3).

**Figure 4.**
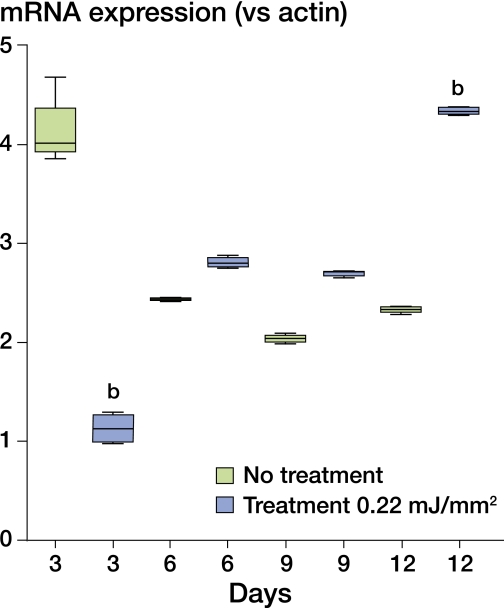
Effect of ESW treatment on collagen type I expression. Relative expression of collagen type I after treatment with ESW (EFD = 0.22mJ/mm^2^, 1,000 impulses) (n = 4). Significance compared to no treatment: **^b^** p < 0.001.

**Figure 5.**
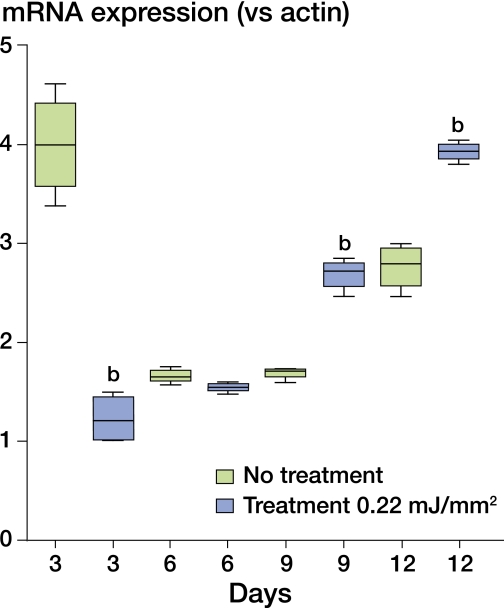
Effect of ESW treatment on collagen type III expression. Relative expression of collagen type III after treatment with ESW (EFD = 0.22mJ/mm^2^, 1,000 impulses) (n = 4). Significance compared to no treatment: **^b^** p < 0.001.

mRNA expression for collagen types I and III showed a different pattern in the untreated and treated groups (p < 0.001): mRNA expression for collagen type I showed a rapid fall in treated fibroblasts relative to the controls (p < 0.001) on the third day of culture and increased again on the following days, with levels higher in treated cells than in untreated cells from the sixth to the twelvth day (p < 0.001) (Figure 4). mRNA expression for collagen type III, after the fall on the third day (p < 0.001), was higher in treated fibroblasts than in untreated fibroblasts on the ninth and the twelvth days (p < 0.001) (Figure 5).

**Figure 6.**
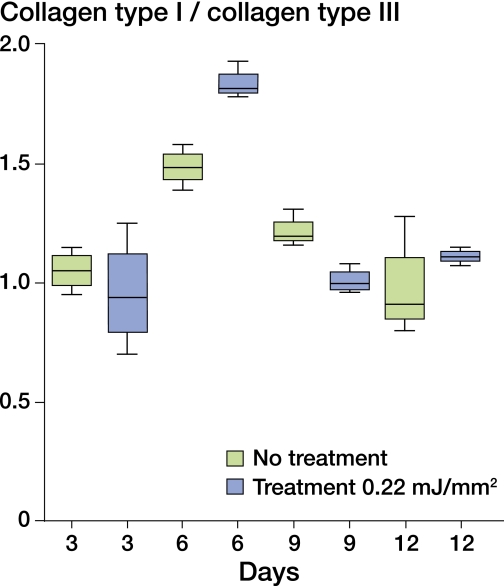
Effect on collagen type I/collagen type III ratio after treatment with ESW (EFD = 0.22mJ/mm^2^, 1,000 impulses) (n = 4).

ESW treatment was associated with an increase in mRNA expression for collagen type I on the sixth day, and anticipated the mRNA expression for collagen type III on the ninth day with respect to the controls (Figure 6).

We compared expression of mRNA for TGF-β1 with the fibroblast growth curve, and similarly mRNA expression for collagen type I and collagen type III, both in untreated and treated cells. The treatment interfered with the behavior of mRNA expression for TGF-β1 versus the other factors considered. The difference between the curves of TGF-β1 mRNA and collagen I mRNA expression in ESW-treated fibroblasts relative to the controls was higher than in all other conditions (linear, p < 0.001: squared, p = 0.005, cubic, p < 0.001). Table I further confirms how the intra-subject variability was conditioned by the time of sampling.

**Table:** Mean values (SD) of cell growth (from absorbance) and relative expression of TGF-β1, collagen type I and collagen type III after treatment with ESW

Day	Treatment	Cell growth (n = 16)	TGFβ (n = 4)	Collagen I (n = 4)	Collagen III (n = 4)
3	No treatment	0.06 (0.004)	3.61 (0.28)	4.14 (0.37)	3.98 (0.54)
	HESW	0.13 (0.003)	2.71 (0.041)	1.15 (0.17)	1.22 (0.26)
6	No treatment	0.15 (0.031)	1.05 (0.13)	2.44 (0.017)	1.64 (0.075)
	HESW	0.24 (0.036)	1.94 (0.052)	2.81 (0.061)	1.53 (0.051)
9	No treatment	0.26 (0.073)	2.60 (0.027)	2.04 (0.043)	1.67 (0.058)
	HESW	0.32 (0.036)	3.50 (0.048)	2.69 (0.031)	2.67 (0.16)
12	No treatment	0.19 (0.044)	3.17 (0.033)	2.32 (0.034)	2.75 (0.24)
	HESW	0.25 (0.030)	3.49 (0.027)	4.33 (0.039)	3.91 (0.10)

## Discussion

We found that shock waves had a dose-dependent destructive effect on cells in suspension, but they also seemed to have a dose-dependent stimulatory effect on cell proliferation. We chose to treat fibroblasts in suspension with low- to medium-energy shock waves; this induced fewer immediate cytodestructive effects and there was a better subsequent stimulation of cell proliferation, in accordance with the work of Wang et al. (2001, 2002) and Martini et al. (2003). Our data indicate that a limited number of shock waves can produce minor damage in soft tissue, favoring the healing process; fibroblast proliferation is one of the main factors and is the first step. Fibroblasts interact with the extracellular matrix and they are influenced by systemic factors related or unrelated to inflammation. Such factors include ischemia, serotonin, endothelin, thrombin, leptin and others (Atamas 2002). Biochemical signals are mediators of the conversion of physical forces into biological activity, and fibroblasts have been shown to be mechanosensitive cells (Iqbal and Zaidi 2005). The proliferative response we observed in treated fibroblasts confirms that ESW treatment imparts a normal mitogenic stimulus.

Many different kinds of cells synthesize TGF-β1, which acts by both autocrine and paracrine mechanisms. It can either stimulate or inhibit proliferation, and either stimulate or inhibit differentiation, thus playing an important role in connective tissue as well as in the healing process, including tendon repair (Atamas 2002). Our findings show that the shock-wave treatment enhances expression of mRNA for TGF-β1 and does not interfere with the normal physiological pattern of expression, over the proliferation curve. The observed decrease in mRNA expression for TGF-β1 on the third day of culture both in untreated and treated fibroblasts is consistent with the injury involved in the transfer of cells onto the wells of the plates and, in treated fibroblasts, even with the ESW treatment, according to the findings of Dahlgreen et al. (2005). The rapid increase both in fibroblast proliferation and in expression of TGF-β1 mRNA is consistent with a strong healing response.

The cavitation effect induced by ESWs results in immediate and long-lasting events at the cellular level, from changes in membrane potential to activation of molecular autocrine and paracrine signals induced by mechanotransduction. The mechanical force of shock waves has been hypothesized to serve as extracellular information that is transmitted to cells and modulates the expression of genes that regulate the growth, function, and differentiation of cells (Chao et al. 2008).

We also found elevated expression of mRNA for collagen types I and III, although with different timing: on the sixth day for collagen type I and on the ninth day for collagen type III. In both cases, ESW treatment enhances expression of the genes encoding collagen types I and III, which is in accordance with the results of other authors who studied human fibroblasts exposed to electromagnetic fields (Rodemann et al. 1989).

The central event in the architecture of tendons is collagen fibrillogenesis (Dahlgreen et al. 2005) and both collagen types I and III have been shown to be key players in the regulation of fibril assembly, which follows the typical row orientation of tendon fibroblasts.

Our data confirm that ESW treatment promotes and improves the repair process through accelerated timing of RNA expression for TGF-β1, collagen I and collagen III (relative to untreated fibroblasts). The results, in line with previous reports using a rat model (Orhan et al. 2004) and a rabbit model (Wang et al. 2008) and also on human tendon ailments (Rompe et al. 2007, 2008), support the efficacy of clinical application of ESW in different types of tendonopathies and tendon injuries.

Since shock-wave treatment circumvents the need for immobilization and for reduced weight bearing (Rompe et al. 2008), it allows—in most cases—a graduated and precise load, favoring the normal remodeling process. Clinical studies have shown the benefit of early mobilization following tendon repair, and several postoperative mobilization protocols have been advocated, as reviewed by Sharma and Maffulli (2005), even if the precise mechanism by which cells respond to load remains to be elucidated. The clinical observations are in accordance with the report by Chen et al. (2004) using a rat model, who described how the increased fibroblast proliferation and biosynthesis of ECM and collagens, as well as the mechanical stress, are crucial for return to normal tendon strength. Most recently, Chao et al (2008) showed that shock waves applied to tenocytes harvested from Spague-Dawley rats can stimulate tenocyte proliferation, which is mediated by early upregulation of proliferating cell nuclear antigen (PCNA) and TGF-β1 gene expression, endogenous NO release, and then TGF-β1 protein and collagen synthesis. The authors hypothesized that shock waves could act as extracellular information that is transmitted to cells, thereby modulating the expression of genes that regulate cell growth, function, and differentiation.

The data from comparison between expression of TGF-β1 mRNA and cell proliferation, and mRNA expression for collagen types I and III suggest that TGF-β1 plays a role in fibroblast growth, which is in accordance with previous publications (Chen et al. 2004, Chao et al. 2008) reporting that ESW increases tenocyte proliferation via TGF-β1. The timing of the increase in mRNA expression for collagen I and collagen III we observed after ESW exposure may also be related to TGF-β1 activation. The differences found in the behavior of TGF-β1 and collagen I mRNA expression in untreated and treated fibroblasts even suggest that different mechanisms may be involved, as suggested by Aaron et al. (2004).

We conclude that ESW treatment applied to fibroblasts in vitro enhances cell proliferation and induces changes in mRNA expression for TGF-β1, collagen I and collagen III that are consistent with activation and acceleration of the healing process. Although the experiments in vitro cannot be directly extrapolated to the in vivo situation, the differences in viability and proliferation rate observed in fibroblasts treated in vitro with different energy and impulse regimes may provide useful information for schedules of ESW treatment in vivo.
